# Substrate Profiling of Tobacco Etch Virus Protease Using a Novel Fluorescence-Assisted Whole-Cell Assay

**DOI:** 10.1371/journal.pone.0016136

**Published:** 2011-01-18

**Authors:** George Kostallas, Per-Åke Löfdahl, Patrik Samuelson

**Affiliations:** Department of Molecular Biotechnology, School of Biotechnology, Royal Institute of Technology (KTH), Stockholm, Sweden; Cardiff University, United Kingdom

## Abstract

Site-specific proteolysis of proteins plays an important role in many cellular functions and is often key to the virulence of infectious organisms. Efficient methods for characterization of proteases and their substrates will therefore help us understand these fundamental processes and thereby hopefully point towards new therapeutic strategies. Here, a novel whole-cell *in vivo* method was used to investigate the substrate preference of the sequence specific tobacco etch virus protease (TEVp). The assay, which utilizes protease-mediated intracellular rescue of genetically encoded short-lived fluorescent substrate reporters to enhance the fluorescence of the entire cell, allowed subtle differences in the processing efficiency of closely related substrate peptides to be detected. Quantitative screening of large combinatorial substrate libraries, through flow cytometry analysis and cell sorting, enabled identification of optimal substrates for TEVp. The peptide, ENLYFQG, identical to the protease's natural substrate peptide, emerged as a strong consensus cleavage sequence, and position P3 (tyrosine, Y) and P1 (glutamine, Q) within the substrate peptide were confirmed as being the most important specificity determinants. In position P1′, glycine (G), serine (S), cysteine (C), alanine (A) and arginine (R) were among the most prevalent residues observed, all known to generate functional TEVp substrates and largely in line with other published studies stating that there is a strong preference for short aliphatic residues in this position. Interestingly, given the complex hydrogen-bonding network that the P6 glutamate (E) is engaged in within the substrate-enzyme complex, an unexpectedly relaxed residue preference was revealed for this position, which has not been reported earlier. Thus, in the light of our results, we believe that our assay, besides enabling protease substrate profiling, also may serve as a highly competitive platform for directed evolution of proteases and their substrates.

## Introduction

Proteases represent one of the largest and most important protein families known, and their importance in processes that govern the life and death of a cell cannot be overestimated. The last decades, it has become evident that proteolysis of bioactive molecules plays an essential role in the regulation of many biological processes, such as signal transduction, RNA-transcription, apoptosis, and development [1], [2]. In addition, proteases are widely used as virulence factors by many infectious microorganisms, viruses and parasites [3]. Consequently, proteases and their substrates are therefore of great interest as potential drug targets. In fact, in humans, proteases represent 5–10% of all drug targets [4], [5].

The function of proteases is regulated either by controlling the spatial and temporal activity or through their ability to discriminate among potential substrates, of which the last is probably the most important mechanism. Accordingly, efficient methods for characterization of proteases and their associated substrates could enhance our understanding of biological systems, which ultimately may result in new therapeutic strategies.

While various biological and chemically based approaches have been developed to study protease substrate specificity and activity [6], [7] they do have their limitations. Many suffer from being insensitive, time consuming, labor intensive, result in incomplete coverage and give no or little information on reaction kinetics. Among the most popular and powerful recent strategies are those based on the use of combinatorial substrate libraries. These libraries can be generated through either biological [8], [9], [10] or chemical means [11], [12]. Collectively, all these methods have been of great importance in determining protease function and specificity. However, innovative high-throughput assays that are accurate and quantitative are still needed; especially when keeping in mind that only a small fraction of all human proteases, encoded by approximately 2% of the human genome, have been studied [13].

With this in mind, we have developed and used a novel label-free high-throughput whole-cell method for quantitative analysis and screening of protease activity *in vivo*, which is presented herein. The essential features of this technology are (i) coexpression of short-lived fluorescent substrates and a protease of interest in the bacterial cytoplasm, (ii) gain-of-fluorescence in proportion to the apparent cleavage efficiency, and (iii) monitoring the whole-cell fluorescence intensity on a flow cytometer.

By applying this methodology, the processing efficiency of closely related substrate peptides could be analyzed for the highly specific tobacco etch virus protease (TEVp). Furthermore, we also adopted this strategy to determine the substrate profile of TEVp through identification of optimal substrates from large genetically encoded substrate libraries. Our results suggest that this methodology may be generally useful for identification and characterization of proteases and their substrate peptides, as well as development of inhibitors and tailor-made substrates. Importantly, it will also open up new possibilities for efficient engineering of proteases towards beneficial properties such as improved activity and desired specificity.

## Results and Discussion

### Substrate processing efficiency analyzed by using a novel fluorescence-assisted whole-cell assay

**Figure 1:**
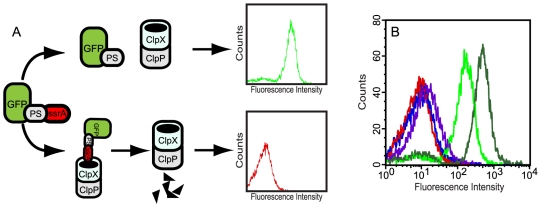
Schematic overview of the fluorescence-assisted whole-cell assay for characterization of proteases. (A) Short-lived fluorescent reporter substrates (GFP-PS-ssrA) and a protease of interest are coexpressed in *E.coli*. Protease-mediated removal of the ssrA-tag from the reporter construct, through *in vivo* processing of the substrate peptide (PS), rescues GFP from cytoplasmic degradation. This enhances the fluorescence intensity of the entire cell, thus enabling flow cytometry analysis and cell sorting to collect desired clones. Explanations: GFP, green fluorescent protein; PS, protease substrate peptide; ssrA, a ClpXP-specific degradation tag. (B) Flow cytometry analysis of *E. coli* cells that coexpress TEVp and different reporters constructs, 2.5 h after induction (0.1 mM IPTG, 0.2% arabinose). Three reporter constructs contained closely related TEVp substrate sequences that only differed in the P1′ position. They are represented by the histograms shown in green (DH5α/pMal-TEV2/pGFP-subG-ssrA^NY^; subG = ENLYFQ**G**), purple (DH5α/pMal-TEV2/pGFP-subV-ssrA^NY^; subV = ENLYFQ**V**) and blue (DH5α/pMal-TEV2/pGFP-subP-ssrA^NY^; subP = ENLYFQ**P**). The negative control (DH5α/pMal-TEV2/pGFP-ssrA^NY^) and the positive control (DH5α/pMal-TEV2/pGFP-subG) are shown in red and dark green, respectively.

We have created a convenient, label-free, and function-based protease assay, suitable for flow cytometry analysis and cell sorting, by utilizing sensitive short-lived fluorescent substrates expressed in *Escherichia coli (E. coli)*. To this end, we used plasmid-encoded ssrA-tagged green fluorescent protein (GFP), containing different substrate peptides (PS) between the GFP and the ssrA-moiety, as a quantitative reporter system. The rationale for this was that (i) the C-terminal ssrA peptide should target the reporter protein, GFP-PS-ssrA, for rapid degradation by the cytoplasmic ClpXP proteolytic machinery [14], [15], [16], unless (ii) coexpression of a substrate specific protease, from an accessory plasmid, catalyzes the removal of the ssrA-tag, thus saving the GFP and enhancing the fluorescence intensity of the entire cell. This can be monitored on a flow cytometer and desired clones isolated through sorting (see Figure 1A for the concept). In our study, we used a modified ssrA-tag (AANDENY**NY**ALAA, ssrA^NY^), containing an extra asparagine (N) and tyrosine (Y) residue (in boldface), since this has been reported to improve ClpXP-mediated degradation of ssrA-tagged proteins [17].

The above-described strategy was used to examine the processing efficiency of the sequence specific tobacco etch virus protease (TEVp) on three closely related substrate reporters that only differed in the P1′ position (the leaving amino acid) of the TEVp substrate peptide, ENLYFQX (where X represents glycine, G; valine, V; or proline, P). The peptides, ENLYFQ**V** (denoted subV) and ENLYFQ**P** (denoted subP), were included in the reporter constructs since they should represent substrates cleaved with low and very low efficiency, respectively, while the natural substrate peptide, ENLYFQ**G** (denoted subG), is one of the best TEVp substrates known [18]. As a positive control, we used cells coexpressing TEVp and GFP-subG (GFP fused to the natural TEVp substrate but lacking the ssrA-tag). Cells that instead expressed substrate-less ssrA-tagged GFP (GFP-ssrA^NY^) together with the protease, constituted a negative control.

When the reporter constructs were expressed alone in *E. coli*, all but the positive control resulted in very low whole-cell fluorescence intensities, as expected (data not shown). The fluorescence intensities were in fact almost identical to the negative control (Figure 1B). However, when TEVp instead was coexpressed with the different reporter constructs, the cells started to fluoresce. Apart from the positive control (GFP-subG), GFP-subG-ssrA^NY^ conferred the highest whole-cell fluorescence intensity while GFP-subV-ssrA^NY^ and GFP-subP-ssrA^NY^ exhibited low and very low fluorescence intensities, respectively (Figure 1B), thus mirroring the substrate processing efficiency previously reported by Kapust et al.[18].

Interestingly, in their study, the peptide ENLYFQV performed relatively well as a substrate (approximately 55% substrate conversion) when it appeared within a fusion protein subjected to intracellular processing by TEVp in *E. coli* cells. However, in two other assays, *in vitro* processing of either fusion proteins or oligopeptides, this peptide proved to be the worst TEVp substrate of all 20 P1′ permutations tested, except ENLYFQP for which no cleavage could be observed at all [18]. In contrast to our method, their *in vivo* assay in general reported much higher cleavage efficiencies than the corresponding *in vitro* experiments, especially for sub-optimal substrates, and was not capable of detecting subtle differences in catalytic turnover.

We believe that the high catalytic efficiencies they observed *in vivo* are probably caused by (i) a relatively high intracellular concentration of protease and fusion substrate (which usually is much higher than in solution) and that (ii) the protease and substrates are constantly exposed to each other. This increases the likelihood of cleavage, and consequently, their *in vivo* results do not correctly reflect the catalytic efficiency obtained in solution. Instead, in our method there is constant competition between the reactions leading to either elimination or accumulation of the fluorescent substrate, which ultimately should result in a more accurate ranking of the substrate processing efficiency, as we see it.

Although GFP-subG-ssrA^NY^ proved to function very well as a TEVp substrate in our system, and in fact was the best among the three ones tested, this particular combination of substrate and protease variant is not necessarily the best there is. Consequently, in order to estimate the theoretical dynamic range of our assay, one must instead use the construct, GFP-subG, which corresponds to the maximal attainable fluorescence signal in the present system configuration. Thus, by comparing the whole-cell fluorescence intensity of the positive control (DH5α/pMal-TEV2/pGFP-subG), with that of the negative control (DH5α/pMal-TEV2/pGFP-ssrA^NY^), the theoretical dynamic range of our assay appeared to be around two orders of magnitude (Figure 1B and Table 1). While GFP-subG-ssrA^NY^ was cleaved efficiently, it did not generate as highly fluorescent cells as the positive control did. Collectively, our results implied that it should be possible to (i) discriminate among substrates exhibiting different processing efficiencies and (ii) potentially, also identify peptides that are cleaved more efficiently than the canonical substrate, ENLYFQG, if such peptides were to exist.

**Table 1:** Incidence and *in vivo* processing efficiency of peptides that emerged from substrate library screening.

	Sequence^1^	Freq.^2^	Perc.^3^	MFI^4^
TEVwt	ENLYFQG			
Library 1	XNLXFXG			
	ENLYFQG	73	41	501
	DNLYFQG	42	23	403
	GNLYFQG	13	7	160
	YNLYFQG	9	5	152
	MNLYFQG	9	5	150
	WNLYFQG	7	4	120
	CNLYFQG	7	4	165
	RNLYFQG	4	2	103
	LNLYFQG	4	2	61
Library 2	XNLXFXX			
	ENLYFQG	143	80	500
	RNLYFQS	14	8	271
	ANLYFQG	4	2	313
	QNLIFQG	2	1	230
	RNLYFQC	2	1	200
Library 3	EXXYXQX			
	ENLYFQG	121	67	512
	ECLYHQG	4	2	311
	ERLYVQM	3	2	227
	ESEYCQE	2	1	209
	EVMYSQA	2	1	178
	EFLYIQD	1	<1	139
	ERGYGQV	1	<1	109
	EVWYCQC	1	<1	107
	EVAYGQK	1	<1	79
	ESRYVQS	1	<1	68
	EGEYWQR	1	<1	57
	ESNYGQM	1	<1	52
Neg. ctrl				10
Pos. ctrl				1075

1. Amino acid sequences.

2. Incidence of a particular clone after screening of library 1, 2 and 3. Data based on 181, 179 and 182 sequenced clones from library 1, 2 and 3, respectively.

3. Frequency percentage of clonal occurrence.

4. Mean fluorescence intensity (au).

### Combinatorial substrate libraries

Despite TEVp's popularity as an efficient reagent for removal of fusion tags from recombinant target proteins [19], [20] and in biomedical research [21], [22], [23], thorough characterization of its substrate specificity by applying combinatorial library approaches have until recently been lacking [24]. Instead, our knowledge of the substrate specificity has largely been based on alignment and comparison of naturally occurring processing sites of TEVp as well as cleavage analysis of substrate variants representing a relatively limited set of amino acid replacements at relevant positions within the TEVp substrate consensus sequence, ENLYFQG [25], [26]. Thus, only a small number of potential substrate sequences have been sampled. To close this gap, we constructed three different genetically encoded combinatorial substrate libraries by using a PCR-based strategy incorporating NNK degenerate codons at relevant positions within the substrate encoding sequence. In the first two libraries, we addressed positions postulated as important specificity determinants [25], [26], [27]. More specifically, in library 1, the positions P6, P3, and P1 were probed (XNLXFXG, Lib1) while library 2 also included P1′ in the randomization process (XNLXFXX, Lib2). In the non-conserved positions (P5, P4 and P2) of naturally occurring TEVp substrates, many different amino acids can be tolerated without total loss of protease processing, albeit generally at reduced rates [25], [26]. In library 3, these positions including P1′ were randomized (EXXYXQX, Lib3).

The libraries had a theoretical diversity of 3.3×10^4^ (pGFP-Lib1-ssrA^NY^) and 1.1×10^6^ gene sequences (pGFP-Lib2-ssrA^NY^ and pGFP-Lib3-ssrA^NY^), whereas the actual libraries contained 5.4×10^5^, 3.1×10^7^, and 3.3×10^7^ colony forming units, respectively. Thus, assuming a random distribution, they are expected to contain all possible “3-mer” and “4-mer” substrate sequences with >99% confidence limits [28]. DNA sequencing of 192 randomly picked colonies from each library revealed no particular sequence bias besides what is imparted by the use of NNK degenerate codons. Moreover, approximately 10.9%, 12.3% and 11.7% of the clones in library 1, 2, and 3, respectively, contained amber stop codons (TAG) due to the applied NNK mutagenesis strategy, which was very close to the theoretical numbers of 9.1% (library 1) and 11.9% (library 2 and library 3).

### Flow cytometry screening for optimal TEVp substrate peptides

**Figure 2:**
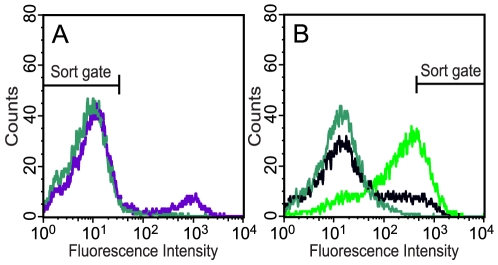
Screening of TEVp substrate libraries. Three different combinatorial substrate libraries were created, using NNK degenerate codons for randomization of position P6, P3, P1 (Lib1); P6, P3, P1, P1′ (Lib2); or P5, P4, P2, P1′ (Lib3) within the cognate TEVp substrate peptide (ENLYFQG). The libraries were then screened for optimal TEVp substrates. (A) Pre-sorting procedure to eliminate false positive clones from the libraries: DH5α/pMal-TEV2/pGFP-Lib1-ssrA^NY^, DH5α/pMal-TEV2/pGFP-Lib2-ssrA^NY^ or DH5α/pMal-TEV2/pGFP-Lib3-ssrA^NY^ cells expressing the substrate libraries alone (i.e., TEVp expression not induced) were analyzed on a flow cytometer and non-fluorescent cells were collected through sorting. Here, the original non-sorted population from library 2 and the corresponding “false-positive depleted” library (after two rounds of sorting) are represented by the histograms in purple and jade, respectively. Library 1 and 3 exhibited the same appearance as library 2 (data not shown). (B) Enrichment-progress when screening the libraries for functional TEVp substrates: The false-positive depleted libraries (see Figure 2A), now coexpressing TEVp and the substrate libraries, were subjected to quantitative flow cytometry analysis, and highly fluorescent cells were collected. The populations from the false-positive depleted library 2, before (jade), after the first (black) and second round of sorting (green) are shown. All three libraries had similar appearance (data not shown).

We then used the fluorescence-assisted whole-cell protease activity assay in a screening procedure to isolate library members harboring substrates processed by TEVp and thereby identify its substrate profile. However, false-positive library members (i.e., clones that express reporter peptides lacking the degradation tag, ssrA, due to stop codons and/or frame-shift mutations, or members where the reporter substrate is processed by an endogenous protease) had first to be depleted from the library cultures. This was done in a pre-sorting experiment by expressing the substrate libraries alone, harvesting non-fluorescent clones through flow cytometry analysis and cell sorting, re-growing them, and then repeating the whole procedure once. As can be seen in Figure 2A, this elimination procedure proved to be very efficient since virtually all false-positive clones were removed. This was later also confirmed when we DNA-sequenced the coselected plasmids from clones that had been isolated in the subsequent screening for functional TEVp substrates, and only five out of 576 sequenced clones proved to contain an amber stop codon within the targeted substrate sequence.

Potentially, the pre-screening could remove some sequence diversity due to endogenous protease activity, and thereby hinder a true identification of preferred substrates. Although we did not analyze the exact nature of the sequences removed, it was comforting to see that the fraction of eliminated cells was close to the actual incidence of amber stop codon-containing clones in the different libraries. This indicates that sequences removed due to the bacteria's own protease activity is likely to be quite few. However, to minimize the need for pre-screening, future substrate libraries should be generated by use of trinucleotide phosphoramidites chemistry (trimer codons) [29], [30], which eliminates the risk of introducing stop codons in the substrate encoding sequence. Off course, false positive clones due to endogenous protease activity may still arise, but such events can readily be identified when the individual clones are analyzed with and without the plasmid encoded protease being expressed. Moreover, should the number of false positive clones be large, they can be eliminated in a final sorting round only collecting low-fluorescent cells when the reporter is expressed alone (*i.e.*, protease expression is not induced).

The pre-sorted libraries were then amplified by growth in liquid cultures before expression of TEVp and the substrate libraries were induced, and the fluorescent clones were collected. After two rounds of sorting, >78% of the enriched clones were highly fluorescent in all three libraries (Figure 2B). The collected library members were plated on LB-agar and then subjected to DNA-sequencing (192 clones from each library), which revealed substrate peptides identical or with high sequence similarity to the canonical substrate, ENLYFQG.

Clones enriched from library 1, in which position P6, P3 and P1 had been probed, only exhibited variance in P6, with a strong preference for the acidic amino acids glutamate (E) and aspartate (D) in 41% and 23% of the clones, respectively (Table 1). Other residues that occupied P6 included glycine (G), tyrosine (Y), methionine (M), tryptophane (W), cysteine (C), and arginine (R) in 7%, 5%, 5%, 4%, 4%, and 2% of the sequenced clones, respectively (Table 1). Position P6 has been postulated to require E in functional substrates [25], and this residue is believed to be involved in an intricate network of hydrogen-bonding interactions in the crystal structure of the enzyme-substrate complex [27]. Therefore, we were surprised to see the relatively relaxed amino acid preference that is reported here. However, in P3 and P1 the amino acid composition were in all cases identical to the wild-type (wt) substrate, namely tyrosine (Y), and glutamine (Q), respectively, thus emphasizing their great significance as specificity determinants.

**Figure 3:**
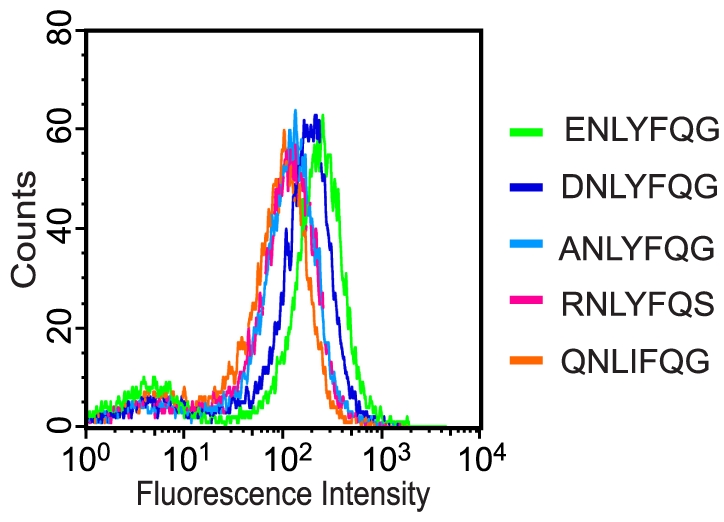
Flow cytometry analysis of individual clones that emerged through screening of library 1 and 2. Each histogram corresponds to a pure culture of DH5α cells coexpressing TEVp and a reporter construct containing a unique substrate peptide, 2.5 h after induction (0.1 mM IPTG, 0.2% arabinose). The different peptides analyzed are indicated in the figure.

Library 2 also resulted in clones expressing substrate peptides with a strong resemblance to the canonical TEVp substrate (Table 1). In fact, after two rounds of sorting, 80% of the clones proved to have peptide sequences identical to the wt substrate (Table 1). Interestingly, the second most frequent substrate peptide (RNLYFQS; 8% of the clones) harbored a positively charged amino acid, arginine (R), in position P6 (Table 1). This was unexpected since library 1 resulted in “winners” with a strong preference for acidic amino acids in that position. However, this particular peptide contained an additional mutation, namely serine (S) in P1′ that is said to improve the catalytic efficiency (*k*_cat_/*K*_M_) by a factor of ∼1.5 compared to the wt substrate with glycine (G) in this position [18]. Thus, this substitution may compensate somewhat for the negative effect of having arginine in P6. Flow cytometry analysis of clones expressing either RNLYFQS or RNLYFQG actually seemed to comply with this, since these peptides resulted in mean fluorescence intensities (MFI) of 271 and 103 arbitrary units (au), respectively (Figure 3 and Table 1).

In general, like library 1, the functional substrate peptides that emerged from library 2 exhibited the greatest variation in P6 that accommodated E, R, G, alanine (A) or glutamine (Q) (Table 1). In position P1′, only G, S or C were detected. This result was to a large extent consistent with previous studies stating that there is a strong preference for short aliphatic amino acids (Gly, Ser, Ala, Met and Cys) in this position [18]. In agreement with the result from library 1, position P3 (Y) and P1 (Q) proved to be very important for substrate functionality as they were completely conserved in all but one peptide in which the tyrosine in P3 was replaced with isoleucine (I) (Table 1).

Although P5 (N), P4 (L, leucine) and P2 (F, phenylalanine), should tolerate a large variety of residue substitutions and still generate a functional substrate [25], [26], we observed a very strong preference for the amino acids found in the canonical substrate. Approximately 67% of the clones from the sorted library 3 actually contained the peptide, ENLYFQG, whereas the rest exhibited quite a diverse range of residues occupying the randomized positions. All enriched clones that were analyzed seemed to contain functional substrates, but none yielded fluorescence intensity larger than the wt substrate peptide (Table 1). Interestingly, among the most prevalent residues in P1′, besides G, were A, S and R, which all are amino acids that should generate functional substrates [18], [24], [25]. Moreover, when we analyzed peptides that were identical in all but the normally non-conserved positions (P5, P4 and P2), they generated different whole-cell fluorescence intensities. Collectively, the results from the analysis of library 3, confirmed that P5, P4 and P2 are not critical specificity determinants but affect the catalytic rate [25], [26]. For a more complete picture of the observed amino acid variability in the targeted positions of library 3, see Figure S1.

With few exceptions, the incidence of a particular peptide among the enriched library population correlated relatively well with its corresponding processing efficiency (Table 1). However, the peptide RNLYFQS (8%) appeared more often than ANLYFQG (2%) and QNLIFQG (1%) despite all being processed with similar efficiency; MFI of 271 au compared to 313 au and 230 au, respectively. Most likely, this deviation is due to sequence bias in the library. For instance, theoretically, genes encoding RNLYFQS are 2.25 times and 4.5 times more frequent than genes coding for ANLYFQG and QNLIFQG, respectively, in library 2.

To our surprise, we did not encounter any clones harboring substrates better than ENLYFQG. For instance, we anticipated ENLYFQS to be detected as it is preferred over ENLYFQG, at least *in vitro*, with a catalytic efficiency (*k_cat_/K_M_*) of 4.51 mM^−1^ s^−1^ versus 3.08 mM^−1^ s^−1^, respectively [18]. However, the amino acids that flanked the substrate peptides were different in our assay and in the study by Kapust et al. [18]. In their experiments, threonine (T) and GTRR, occupied P7 and P2′P3′P4′P5′, respectively, while our reporter constructs instead harbored D and VDAA in the corresponding positions. This difference may have affected the substrate processing efficiency in such a way that ENLYFQS is not necessarily the best TEVp substrate in our reporter system.

Another conceivable explanation for the absence of this particular peptide is that it for some reason was not included (or sampled) in library 2 and 3, although this seems unlikely since they were large enough to theoretically include all possible gene sequences with more than 99% probability. Alternatively, incomplete repression of the TEVp expression during the pre-sorting procedure may have caused elevated fluorescence intensity in cells containing highly efficient substrate peptides. Such cells, potentially including ENLYFQS, would then have been considered as false-positives and not collected.

Nevertheless, our results suggest that ENLYFQG is the best, or at least preferred, TEVp substrate. There is also support for this observation in a very recent study by Boulware et al. [24], where cell libraries of surface-displayed random peptides were incubated with exogenous TEVp and screened for TEVp substrates. The substrates that they identified showed high sequence similarity to the native substrate, and in fact, seven out of the eight best peptides harbored G instead of S in P1′ [24]. However, we cannot rule out the possibility that some other peptide sequence can be processed faster than ENLYFQG.

### Characterization of substrate hydrolysis *in vivo* and *in vitro*

The use of protease-mediated rescue of GFP allowed for a relatively straightforward characterization of the substrate processing efficiency. Several individual clones from the sorted libraries, representing different substrate peptide sequences, were subjected to flow cytometry analysis in order to rank them on the basis of their whole-cell fluorescence intensity. The most common substrate peptides (ENLYFQG and DNLYFQG) from library 1 resulted in MFI of 501 and 403 au, respectively (Figure 3 and Table 1). The other peptides that were assayed yielded MFI ranging from approximately 60 to 165 au (Table 1). Individual clones, isolated from library 2, were analyzed in the same fashion, and besides the canonical substrate peptide (ENLYFQG), also RNLYFQS, ANLYFQG, QNLIFQG, and RNLYFQC appeared to be functional substrates, resulting in MFI of 271, 313, 230, and 200 au, respectively (Figure 3 and Table 1).

Although it is difficult to directly compare quantitative data obtained through different methods, it has not escaped our notice that the hierarchical order of the processing efficiency of the different substrate peptides to a large extent agreed with previous findings by Dougherty et al [25]. For instance, when they investigated the preferred amino acid composition in position P6, the order proved to be E>Q>G>M>A>L, which was the same as for our whole-cell fluorescence data (Table 1) on substrate peptides that were similar in both studies. However, in one instance, when the peptide contained alanine in P6 it performed better in our assay.

**Figure 4:**
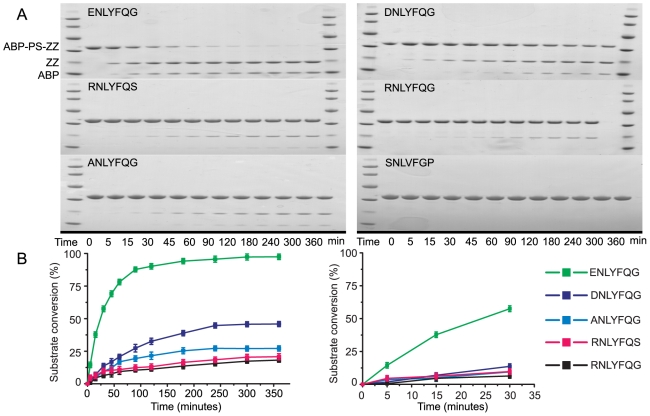
Substrate conversion of different ABP-PS-ZZ fusion proteins by recombinantly produced TEVp. (A) SDS-PAGE analysis (representative of three independent experiments). ABP-PS-ZZ (10 µg) was incubated with TEVp (0.3 µg) in TEVp reaction buffer at 37°C for different incubation times (0, 5 min, 15 min, 30 min, 45 min, 60 min, 90 min, 2 h, 3 h, 4 h, 5 h and 6 h). The substrate peptide sequence (PS) in each analyzed fusion protein is indicated in the figure. As a negative control, we used a fusion protein containing a peptide (SNLVFGP) that cannot be cleaved by TEVp. (B) The substrate conversion of each given fusion protein plotted against time based on densitometric analysis of the SDS-PAGE gels in Figure 4A.

Furthermore, to find out how our hydrolysis data, obtained through flow cytometry analysis on pure cultures expressing various reporter substrates, relates to those measured *in vitro*, we analyzed the cleavage kinetics for soluble fusion proteins containing the corresponding substrate peptides. We reasoned that this should be a justified analysis since TEVp is frequently used to remove affinity tags from fusion proteins. A range of substrates, each composed of ABP-PS-ZZ, were expressed in *E. coli* and purified on affinity chromatography columns; PS represents different TEVp substrate peptides while the albumin binding protein (ABP) and IgG-binding protein Z, are two highly soluble affinity tags derived from streptococcal protein G and staphylococcal protein A, respectively [31]. Processing of the various polypeptide substrates by TEVp was analyzed over time using polyacrylamide gel electrophoresis (Figure 4A) and the substrate conversion at each time point was calculated and plotted (Figure 4B). Cleavage was rapid and close to 100% (98%) efficient for the fusion protein containing the usual TEVp substrate peptide (ENLYFQG) over the 6 h incubation time (Figure 4A and 4B). For the other substrate variants that were tested, processing proved to be less efficient: 46% (DNLYFQG), 27% (ANLYFQG), 21% (RNLYFQS), and 18% (RNLYFQG). In addition, determining the conversion of each substrate at halftime was also used for comparing the effect of the substrate sequence on cleavage; halftime is the time point, at which 50% of the fusion protein containing the wt substrate peptide had been processed, in this particular case approximately 23.5 minutes. At this time point, only 11%, 7%, 8%, and 6% of the respective substrates had been processed (Figure 4B).

Although there is not a perfect correlation between data obtained from the cell-based *in vivo* fluorescence assay and the *in vitro* substrate conversion experiment, importantly, the hierarchical order of the substrate processing efficiency was identical in either context.

### Conclusions

To summarize, we have presented a novel and powerful method that allowed us to quantitatively and qualitatively analyze protease substrate processing *in vivo* and also rapidly identify the substrate preference of TEVp. In our search for highly efficient TEVp substrates, the peptide ENLYFQG, identical to the protease's natural substrate peptide, emerged as a strong consensus cleavage sequence. However, we did not encounter any other peptide that performed better, although the dynamic range provided by our system should have allowed for that. This may suggest that this particular combination of substrate peptide and protease variant has coevolved to function optimally together. Overall, our findings were to a large degree consistent with previously reported studies. For instance, position P3 (Y) and P1 (Q) were almost completely invariant in all functional substrates, thereby confirming them as being the most important specificity determinants. Moreover, in position P1′, glycine (G), serine (S), cysteine (C), alanine (A) and arginine (R) were among the most prevalent residues observed, all known to generate functional TEVp substrates and in line with other studies stating that there is a strong preference for short aliphatic residues in this position. Remarkably, we noticed that a diverse range of amino acids (E, D, A, R, Q, G, Y, M, W, C, and L) could be accommodated in P6 and still generate functional substrates. This came as a surprise since the glutamate that normally occupies this position is believed to participate in an intricate hydrogen-bonding network in the protease-substrate complex [27], and has been postulated as one of the most important specificity determinants [26], [27]. The fact that TEVp appears to have a larger potential substrate repertoire than previously known may have impact on its use in biomedical research and industry, where its popularity is based on its high sequence specificity and activity.

Although the method proved useful for identification of substrate peptides, the exact cut position cannot be determined directly with our assay, as also is the case with many competing methods for substrate identification. While this might not be an issue for a well studied protease like TEVp whose substrate profile is well know, this can be an issue for less-characterized proteases. One way to determine the scissile bond is through mass spectrometry (MS) analysis of cleavage products from synthetic peptide substrates [32], [33]. However, we believe that our system also is amendable to MS-based identification of the exact cleavage site. In such a case, the intracellular protein content from bacteria coexpressing substrate-containing reporters (GFP-PS-ssrA) and protease would first be released and the cleaved reporter then affinity captured specifically, before being analyzed by MS (or MS-MS) to reveal the cleavage position. This approach would thus eliminate the need for synthetic peptide substrates whose synthesis can be difficult, costly and time consuming.

Indeed, there are other methods that also utilize or could utilize competing substrate peptides for screening or selection purposes; either biological systems such as substrate phage [9], cell displayed libraries of peptide substrates [10], and protease sensitive genetic screen based on cyclic AMP signaling cascade in *E. coli* [34] for example, or chemical methods based on various synthetic peptide library formats [35], but none of them operate in the same way as our system does. For example, unlike most other methods, there is no need for production and purification of the investigated protease and/or synthetic substrates, which otherwise can be time consuming, complicated and expensive. Moreover, besides the high sensitivity and relatively large dynamic range provided by our system, it enables direct quantitative measurement of the substrate conversion and selective enrichment of clones with a desired processing efficiency. These features should make the presented method particularly attractive for the engineering of substrate peptides exhibiting a defined catalytic turnover that could be of interest in the construction of synthetic and regulatory circuits, prodrug design, inhibitor discovery, and substrate probe development. Finally, our system is unique in that it allows for the identification of substrate peptides as well as directed evolution of proteases in such a straightforward way, which is due to the strong and simple genotype-phenotype link (the protease and substrate reporter are plasmid-encoded and coexpressed within the analyzed cells).

Thus, in the light of our results and what has been discussed here, we believe that our methodology holds great promise as a highly competitive platform for engineering of proteases and their substrates, rapid substrate profiling, and protease inhibitor development, which all are subjects of biological, biomedical and industrial relevance.

## Materials and Methods

### Bacterial strains and reagents

*E. coli* strain RR1ΔM15 [36] was used as host during construction of plasmids. *E. coli* strain DH5α (Gibco) was used for flow cytometry analysis and cell sorting. *E. coli* strain Rosetta (DE3)pLysS (Novagen) was used for production of ABP-PS-ZZ and TEVp. Culture media and chemicals were from Merck and Sigma-Aldrich, respectively. DNA modifying enzymes were all from New England Biolabs and used according to the manufacturer's recommendations. Primers were purchased from MWG Biotech (for a list of all oligonucleotides used in this study, see Table S1).

### Plasmid constructions

For an overview of the relevant important plasmids constructed and used in this study, see Figure S2.

To create a TEVp variant suitable for recombinant expression in *E. coli* we had the autoproteolysis site inactivated (S219V) [37], [38] and several rare arginine codons exchanged for more common ones. This was done by combining relevant regions of the TEVp-encoding vectors pRK693 [39] and pRK793 [37] through gene splicing by overlap extension [40]. More specifically, two separate PCR reactions were performed, using the primer pairs SAPA60/SAPA73, and SAPA72/SAPA61, with pRK693 and pRK793 as templates, respectively. The two amplicons were then purified, mixed and used as template in a third PCR reaction, with primers SAPA60 and SAPA61, to generate the PCR-spliced full-length product. This product was digested with *Hin*dIII as well as *Bam*HI and ligated into the *Hin*dIII/*Bam*HI-digested backbone of pRK693, yielding pMal-TEV1. TEVp, expressed recombinantly without any solubility-promoting fusion tag exhibits very poor solubility *in vivo* [41], [42]. To alleviate this, a sequence encoding the maltose binding protein (MBP), including a C-terminally attached TEVp substrate peptide, ENLYFQG, was transferred as a *Hin*dIII/*Mlu*I-fragment from pRK793 into the *Hin*dIII/*Mlu*I-digested backbone of pMal-TEV1, resulting in pMal-TEV2. Thus, when TEVp is expressed from pMal-TEV2, the protease will not be permanently fused to the solubility enhancing MBP-moiety since this domain is cleaved off in the cell by the protease itself [41].

Several different TEVp substrate reporter plasmids were constructed from pGFP-ssrA^NY^, which encodes GFP fused to a modified ssrA-tag (AANDENY**NY**ALAA, ssrA^NY^) containing an extra asparagine and tyrosine residue (in boldface) [17], which targets the GFP for efficient destruction by the cytoplasmic degradation complex, ClpXP [15], [43]. To this end, GFPmut3 [44] was PCR-amplified using primers SAPA46 and SAPA47, digested with *Sac*I and *Hin*dIII and then ligated into *Sac*I/*Hin*dIII-digested pBAD33 [45], yielding pGFP-ssrA. From this plasmid we created pGFP-ssrA^NY^ by using a QuikChange Site-Directed Mutagenesis Kit (Stratagene) together with primers GEKO14 and GEKO15.

The primer pairs SAPA62/SAPA63, SAPA65/SAPA65, SAPA66/SAPA67, and SAPA68/SAPA69 were used to generate linkers encoding different substrate peptides. The first three, encoded TEVp substrate variants in which the P1′ position of the wt substrate peptide (ENLYFQG) either accommodated glycine (G), valine (V) or proline (P), respectively. The last linker encoded the wt substrate peptide directly followed by a stop codon. All linkers were inserted into *Sal*I-digested pGFP-ssrA^NY^ to create pGFP-subG-ssrA^NY^, pGFP-subV-ssrA^NY^, pGFP-subP-ssrA^NY^ and pGFP-subG, respectively.

For production of soluble fusion proteins (ABP-PS-ZZ), containing different TEVp substrate peptides (PS), plasmid pABP-PS_WT_-ZZ was constructed. First, primers zz-for_1 and zz-rev were employed for amplification of the ZZ gene fragment from pEZZ-cutinase [46]. The resulting amplicon was used as template in a second PCR reaction with primers zz-for_2 and zz-rev. The final PCR product, encoding the TEVp wt cleavage site (ENLYFQG) fused to ZZ, was gel purified, digested with *Bam*HI and *Eco*RI, and ligated into *Bam*HI/*Eco*RI-digested pAff8c [47], yielding pABP-PS_WT_-ZZ. Several pABP-PS-ZZ-variants, encoding substrate peptides other than the TEVp wt substrate, were also created by insertion of different relevant oligonucleotide linkers into *Bam*HI/*Pst*I-digested pABP-PS_WT_-ZZ.

In order to generate a vector for production of TEVp, a *Kpn*I-site that should facilitate the replacement of TEVp with other proteases of interest was first introduced upstream of the TEVp coding sequence in pMal-TEV2. This was done by PCR amplification of the plasmid pMal-TEV2 using primers TEV_mut_fw and TEV_mut_rv. The template plasmid was then degraded through addition of *Dpn*I. The remaining PCR product was cleaned using a QiaQuick PCR clean–up kit (Qiagen), digested with *Kpn*I, and finally recircularized with T4 DNA ligase to generate pMal-TEV3. The MBP-TEV gene fragment from plasmid pMal-TEV3 was PCR amplified in two separate PCR reactions, one with primers Mal1 and Mal3 and another with Mal2 and Mal4. The resulting amplicons were mixed in equimolar amounts and combined in a hybridization reaction by first raising the temperature to 95°C followed by slow cooling to room temperature. After phosphorylation of the hybridized “sticky end” product, it was ligated downstream of the T7 promoter into *Bam*HI/*Nco*I-digested pAff8c, resulting in the TEVp production vector, pTEVprod.

All plasmid constructs were verified by standard DNA sequencing using Big Dye Terminator 3.1 Cycle Sequencing Kit and ABI Prisma 3700 sequencer (Applied Biosystems).

### Library constructions

Two different combinatorial TEVp substrate libraries, in the form of XNLXFXG and XNLXFXX, respectively, where X is any amino acid, were constructed by using a PCR-based strategy. For construction of library 1, plasmid pGFP-subG-ssrA^NY^ was used as template in a PCR reaction with oligonucleotide GEKO19 and the randomized primer GEKOLib1. GEKOLib1 introduced NNK degenerate codons at positions corresponding to the P6, P3 and P1 residues of the TEVp wt substrate located between the coding sequences for GFP and ssrA^NY^. The amplified gene fragment was purified by preparative agarose gel electrophoresis, digested with *Sal*I and *Dra*III, gel-purified a second time, and then ligated into *Sal*I/*Dra*III-digested pGFP-ssrA^NY^, resulting in pGFP-Lib1-ssrA^NY^. The library ligation mixture (pGFP-Lib1-ssrA^NY^) was transformed into chemically competent DH5α cells [48] that already harbored the TEVp expression vector pMal-TEV2. Two analogous libraries, pGFP-Lib2-ssrA^NY^ and pGFP-Lib3-ssrA^NY^, were constructed in a similar way. This time, the GEKOLib2 primer was utilized to randomize the TEVp substrate peptide in positions P6, P3, P1, and P1′, while GEKOLib3 instead addressed P5, P4, P2, and P1′. The library sizes were determined by plating serial dilutions of the transformed cell suspensions on LB agar plates containing 100 µg/ml ampicillin and 20 µg/ml chloramphenicol. All libraries were stored at −80°C as cell suspensions in LB broth supplemented with glycerol (15% final concentration) until screened. The library quality, with respect to sequence variation and frequency of undesired mutations such as nucleotide deletions and insertions etc, was checked by DNA sequencing of 192 randomly picked clones from each library.

### Flow cytometry analysis and library screening

For individual clone analysis and library screening, overnight cultures of DH5α cells, harboring the TEVp expression vector, pMal-TEV2, and a relevant TEVp substrate reporter vector (for example pGFP-subG-ssrA^NY^, pGFP-ssrA^NY^ or pGFP-Lib1-ssrA^NY^; for an overview of other suitable reporter plasmids, see Figure S1), were subcultured by dilution (1∶75 for clone analysis and 1∶150 for the libraries) into fresh LB broth containing 100 µg/ml ampicillin and 20 µg/ml chloramphenicol, and incubated at 37°C in a rotary shaker set at 150 rpm. When the cultures reached a cell density of OD_600_≈0.5, IPTG was added to a final concentration of 0.1 mM to initiate TEVp expression. The cultures were now placed in a shaker set at 30°C, 150 rpm, and after 30 minutes, expression of the reporter constructs were induced by adding L(+)-arabinose to a final concentration of 0.2%. Two hours later, 1 ml of each culture was placed on ice and 5–10 µl from each sample was diluted with 1 ml ice-cold 1×PBS (11,68 g NaCl; 9,44 g Na_2_HPO_4_; 5,28 g NaH_2_PO_4_·2H_2_O; 1,000 ml MilliQ purified water; pH 7,2) and kept on ice until analyzed on a FACSVantage SE flow cytometer (Becton Dickinson). The throughput rate for the analysis was 300 events/sec with 488 nm excitation wavelength (argon ion laser), emission detection between 510 and 530 nm, and 10,000 events were recorded for each sample.

Library samples were prepared for fluorescence-activated cell sorting in essentially the same way as for flow cytometry analysis of individual clones. The amount of thawed cells (DH5α/pMal-TEV2/pGFP-Lib1-ssrA^NY^, DH5α/pMal-TEV2/pGFP-Lib2-ssrA^NY^ or DH5α/pMal-TEV2/pGFP-Lib3-ssrA^NY^) used for the overnight inoculation corresponded to at least tenfold the size of each library. Right before the library screening, a small aliquot of each culture was diluted 100-fold in ice-cold 1×PBS. The cells were then analyzed on a FACSVantage SE flow cytometer with a throughput rate of 250–400 events/s and sorted according to desired fluorescence intensity criteria directly into LB media. After sorting, the collected cell suspensions were either plated on solid LB agar containing the appropriate antibiotics or re-grown overnight for further rounds of analysis and cell sorting. More specifically, first we conducted two initial rounds of sorting that aimed at removing false-positive clones from the libraries (i.e., cells expressing randomized substrate genes containing stop codons and frame-shift mutations thereby lacking the degradation tag, ssrA^NY^, or members where the reporter substrate could be processed by an endogenous protease). This was accomplished by collecting non-fluorescent cells from library cultures that expressed the substrate reporters alone (only 0.2% arabinose and no IPTG was added to the cell cultures). The resulting sorted library population was then amplified by growth in liquid cultures before expression of both TEVp and reporter substrate was induced. Highly fluorescent clones, resulting from TEVp-mediated substrate processing, were collected through sorting. After two rounds, DNA sequencing of 192 randomly picked colonies from each enriched library population enabled the identification of functional TEVp substrate peptides. Clones that either occurred often or exhibited interesting substrate sequence pattern were selected for further flow cytometry analysis to score the substrate processing efficiency.

### Protein expression and purification

*E. coli* Rosetta (DE3)pLysS/pTEVprod, was cultured overnight in a rotary shaker at 30°C, 150 rpm in 500 ml tryptic soy broth (TSB) supplemented with 50 µg/ml kanamycin and 20 µg/ml chloramphenicol. Five milliliters of the overnight culture was re-inoculated in 500 ml fresh TSB medium containing the same antibiotics and left to grow at 30°C, 150 rpm until OD_600_ reached 0.5. At that point, TEVp expression was induced by adding IPTG (0.5 mM final concentration). The cultures were left to grow for another four hours at 30°C, 150 rpm before the cells were harvested by centrifugation (2,500×*g*, 10 min) and resuspended in 60 ml TALON buffer (50 mM NaH_2_PO_4_, 300 NaCl, pH 7.5). Cells lysis was achieved through sonication (4 min, 40% effect) and the resulting cell debris was removed by centrifugation (30 min, 40,000×*g*). The soluble protein fraction (supernatant) was loaded on a 2 ml TALON metal affinity column (Clontech) and treated according to the manufacturer's recommendations. For elution of the immobilized TEVp, the column was washed with 5×1ml 1.5 M imidazole (pH 7). Notice that although TEVp is originally expressed as MBP-ENLYFQG-His_6_-TEVp, the final protease product will be devoid of the solubility enhancing MBP-moiety including the majority of the substrate peptide since they are detached by the protease itself in the cytoplasm. The eluted fractions, containing the target protein, were pooled, followed by a buffer exchange into TEVp reaction buffer (100 mM Tris-HCl (pH 8.0), 25 mM NaCl, 0.5 mM EDTA, 1 mM DTT) using a PD-10 desalting column (Amersham). The quality of the purified TEVp was analyzed by sodium dodecyl sulphate-polyacrylamide gel electrophoresis (SDS-PAGE) and subsequent staining with GelCode Blue Stain Reagent (Pierce). The protease was then used in an *in vitro* assay to analyze the conversion of various recombinant substrate proteins.

Expression of the different ABP-PS-ZZ protein variants was performed essentially as described above (where PS represents alternative TEVp substrate peptides, and ABP is an albumin binding protein derived from streptococcal protein G, while Z constitutes an IgG-binding staphylococcal protein A-derivative [31]. However, the harvested cells (*E. coli* Rosetta (DE3)pLysS/pABP-PS-ZZ) were resuspended in 20 ml denaturing lysis buffer (7 M guanidium chloride, 47 mM Na_2_HPO_4_, 2.67 mM NaH_2_PO_4_, 10 mM Tris-HCl, 100 mM NaCl, 20 mM β-mercaptoethanol, pH 8.0) and incubated for 2h at 37°C. The cell lysate was centrifuged at 35,300×*g* for 30 minutes and the supernatant loaded on an ASPEC XL4 (Gilson) automated protein purification system equipped with columns filled with 1 mL Talon metal affinity chromatography resin (Clontech), and purified according to the protocol described by Steen et al. [49]. The buffer of the purified protein fractions was exchanged into TEVp reaction buffer by using PD-10 desalting columns.

### In vitro cleavage of different fusion substrates

Enzymatic reactions comprising 10 µg of recombinant ABP-PS-ZZ fusion proteins (containing different substrate peptides) and 0.3 µg recombinant TEVp in 30 µl TEVp reaction buffer (100 mM Tris-HCl (pH 8.0), 25 mM NaCl, 0.5 mM EDTA, 1 mM DTT) were incubated at 37°C for different incubation times (0, 5 min, 15 min, 30 min, 45 min, 60 min, 90 min, 2 h, 3 h, 4 h, 5 h and 6 h). The reactions were terminated by adding 3.3 µl 1% SDS to a final concentration of 0.1%. Before heating at 96°C for 7 min, 10 µl of 3×SDS denaturing buffer (150 mM TRIS, 300 mM DTT, 6% SDS, 0.3% bromophenol blue and 30% glycerol) was added to 20 µl of the reactions. After denaturation, the samples were analyzed on an SDS-PAGE gel (Novex 4–12% Tris-glycine gradient gel, Invitrogen) and then stained using GelCode Blue Stain Reagent (Pierce). Finally, the visualized protein bands were quantified by densitometric means using Quantity One 1-D Analysis Software (Bio-Rad). All experiments were executed in three independent replicates.

## Supporting Information

Figure S1 **Sequence variance of substrate peptides that emerged from library 3.** Sequence logo (http://weblogo.berkeley.edu/) based on functional substrate sequences that emerged from the screening of library 3. The dominant sequence ENLYFQG (approximately 67% of the clones harbored this peptide) was excluded to be able to reveal the “residual” sequence variance for the addressed positions (P5, P4, P2 and P1′). The incidence of each amino acid is proportional to the height of the corresponding letter at that position.
(EPS)

Figure S2 **Plasmid chart showing the important vectors constructed and used in this study.**
(EPS)

Table S1 **Oligonucleotides used in this study.**
(DOC)
