# Supplementary material for: Substrate Profiling of Tobacco Etch Virus Protease Using a Novel Fluorescence-Assisted Whole-Cell Assay
Source: PLoS One. 2011 Jan 18;6(1):e16136. doi: 10.1371/journal.pone.0016136 (PMC3022733; doi:10.1371/journal.pone.0016136)
Supplement: Table S1 — Oligonucleotides used in this study. (DOC) [file pone.0016136.s003.doc]

| Primer | Sequence (5’-3’) |
| --- | --- |
| GEKO14 | gcagcaaacgacgaaaactacaactacgctttagcagcttaa |
| GEKO15 | ttaagctgctaaagcgtagttgtagttttcgtcgtttgctgc |
| GEKO19 | ggcttaccatctggccccagtgctgcaatgatacc |
| GEKOLIB1 | caaagtcgacnnkaacctgnnkttcnnkggtgtcgatgcagcaaacgacgaaaactac |
| GEKOLIB2 | caaagtcgacnnkaacctgnnkttcnnknnkgtcgatgcagcaaacgacgaaaactac |
| GEKOLIB3 | caaagtcgacgaannknnktacnnkcagnnkgtcgatgcagcaaacgacgaaaactac |
| Mal1 | catgaaaatcgaagaaggtaaactgg |
| Mal2 | aaaatcgaagaaggtaaactgg |
| Mal3 | cttattagcgacggcgacgac |
| Mal4 | gatccttattagcgacggcgacgac |
| TEV_mut_fw | ggggtacccatcatcatcatcatcatcatggag |
| TEV_mut_rv | ggggtaccttgaaaataaagattttctccccttc |
| SAPA46 | cgaattcgagctcgaattctctagattaaagaggagaaaggtacccatgagtaaaggagaagaacttttcactggag |
| SAPA47 | aaaacagccaagcttgcatgcttaagctgctaaagcgtagttttcgtcgtttgctgcgtcgactttgtatagttcatccatgccatgtgtaatcc |
| SAPA60 | tcgatgaagccctgaaagacg |
| SAPA61 | ggcgattaagttgggtaacgc |
| SAPA62 | tcgacgaaaacctgtacttccagggtg |
| SAPA63 | tcgacaccctggaagtacaggttttcg |
| SAPA64 | tcgacgaaaacctgtacttccaggtgg |
| SAPA65 | tcgaccacctggaagtacaggttttcg |
| SAPA66 | tcgacgaaaacctgtacttccagccgg |
| SAPA67 | tcgaccggctggaagtacaggttttcg |
| SAPA68 | tcgacgaaaacctgtacttccagggttaag |
| SAPA69 | tcgacttaaccctggaagtacaggttttcg |
| SAPA72 | gttggtatacactcagcatcg |
| SAPA73 | cgatgctgagtgtataccaac |
| zz-for_1 | tacttccagggtcacgatgaagccgtagacaac |
| zz-for_2 | cgggatccggaaaacctgtacttccagggt |
| zz-rev | acgaattcgcgtcttatttcggcgcctg |
